# Incidence, Risk Factors, and Nomogram of Transfusion and Associated Complications in Nonfracture Patients following Total Hip Arthroplasty

**DOI:** 10.1155/2020/2928945

**Published:** 2020-10-14

**Authors:** Yuanhe Wang, Cui Wang, Chuan Hu, Bo Chen, Jianyi Li, Yongming Xi

**Affiliations:** ^1^Department of Orthopaedic Surgery, the Affiliated Hospital of Qingdao University, Qingdao 266071, China; ^2^Wenzhou Medical University, Zhejiang 325000, China

## Abstract

The incidence, risk factors, and associated complications of perioperative transfusion in nonfracture patients following total hip arthroplasty (THA) are unclear. The aim of the present research was to study the predictors of transfusion risk in nonfracture patients following THA and develop a nomogram. One thousand six hundred and thirty-five patients who underwent THA due to nonfracture disease in our institution between September 2013 and July 2017 were included. Independent predictors of transfusion were identified by univariate, LASSO, and multivariate analyses. A nomogram was established based on independent predictors. In addition, a prospective cohort was used to validate the nomogram. The area under the receiver operating characteristic curve was utilized to evaluate the discrimination of the nomogram. Calibration and decision curve analyses were established to evaluate the nomogram. In addition, the association between perioperative transfusion and 30- and 90-day complications was studied. The incidence of transfusion was 15.78%, and 10 independent predictors were confirmed. The areas under the curve of the nomogram were 0.834 and 0.867 in the training and validation cohorts, respectively. Moreover, the area under the curve of the nomogram was significantly higher than that of any single predictor in both the training and validation cohorts. Calibration curve and decision curve analyses in both the training and validation cohorts showed good performance of the nomogram. In addition, perioperative transfusion was identified as an independent risk factor for both 30- and 90-day complications. Generally, ten transfusion-related factors for nonfracture patients following THA were identified. A validated nomogram was established, and several adverse events were confirmed to be associated with transfusion.

## 1. Introduction

Total hip arthroplasty (THA) is a cost-effective procedure for the treatment of hip diseases, and the outcome has been confirmed in previous studies. However, due to blood loss, some patients receive allogenic blood transfusion during the perioperative period. Previous studies have shown that the incidence of blood transfusion in THA patients ranges from 16.9% to 50% [1–3]. However, transfusion not only increases the length of hospital stay and costs but also increases the risk of complications and mortality [3–9]. Therefore, blood management is crucial to the positive prognosis of THA patients, and preventive measures are needed to reduce the incidence of transfusion to reduce the risk of adverse events.

Previous studies have shown that older age, female sex, longer operation time, lower preoperative hemoglobin levels, and higher ASA class are risk factors for perioperative transfusion after THA [5, 10–12]. However, the indications of THA include hip fracture and nonhip fracture. The former includes femoral neck fracture, intertrochanteric fracture, and femoral head fracture, while the latter includes avascular necrosis of femoral head (ANFH), hip osteoarthritis (OA), developmental dysplasia of the hip (DDH), and other nonfracture diseases of the hip. Most previous studies that focused on the incidence, risk factors, or associated complications of transfusion in patients undergoing THA combined hip fracture and nonhip fracture patients or only analyzed patients with hip fractures; no specific study has focused on nonfracture patients [13–15]. In addition, recent studies have shown that there are different outcomes between hip fracture and nonfracture patients after THA [16–18]. Therefore, there is an urgent need to study the perioperative hematological management of nonfracture patients.

Therefore, the aim of the present study was to determine the incidence and risk factors for perioperative blood transfusion, develop a nomogram to predict the perioperative transfusion risk, and identify the relationship between perioperative blood transfusion and postoperative complications after THA in nonhip fracture patients.

## 2. Materials and Methods

Patients who underwent THA due to nonhip fracture diseases from September 2013 to July 2017 in our institution were reviewed. Patients with incomplete data, patients with a history of coagulation disorders, and patients who received allogenic blood transfusion within 30 days before THA were excluded. Finally, a total of 1635 patients were enrolled as follows: 1282 patients with ANFH, 162 patients with OA, 169 patients with DDH, 8 patients with rheumatoid arthritis (RA), 9 patients with ankylosing spondylitis (AS), and 5 patients with other diseases. Moreover, from August 2017 to May 2019, we prospectively included patients who underwent THA in our hospital with the same inclusion and exclusion criteria to create the validation cohort. All procedures of this study were conducted in compliance with the Declaration of Helsinki and were reviewed and approved by the Ethics Committee of the Affiliated Hospital of Qingdao University (number: QYFY WZLL 25927).

Data were obtained from the electronic medical record system of our hospital. Baseline data (age, gender, height, weight, body mass index (BMI), smoking, alcoholism, length of hospital stay, and total in-hospital costs), preoperative comorbidity data (Charlson comorbidity index (CCI), hypertension, diabetes, coronary heart disease, cerebrovascular disease, arrhythmia, respiratory disease, digestive system disease, urinary system disease, mental disease, and uterine fibroids), operative data (indication, anesthesia, operation time, procedures, approach, drainage, and tranexamic acid (TXA) use), preoperative laboratory test results (blood type of ABO, preoperative hemoglobin (Hb) level, preoperative hematocrit (HCT), preoperative red cell distribution width (RDW), preoperative white blood cell (WBC), preoperative platelet (PLT) and preoperative reticulocyte (RET)), and follow-up data were collected. Follow-up data included blood transfusion within 14 days after surgery, 30-day complications, and 90-day complications.

The primary outcome of our study was perioperative blood transfusion, which was defined as patients who received allogenic blood transfusion within 14 days after THA. Moreover, it should be pointed out that the indication for transfusion in our institution is patients with Hb < 70 g/L or patients with Hb < 80 g/L but with symptoms of anemia. The second outcomes were 30- and 90-day complications, which were identified as patients who experienced complications within 30 days or 90 days after THA, respectively. Complications included hematoma, surgical site infection, periprosthetic joint infection, periprosthetic femoral fracture, dislocation, aseptic loosening, shock, myocardial infarction, heart failure, pulmonary embolism, cerebral infarction, delirium, acute stress ulcer, arrhythmia, acute renal injury, deep vein thrombosis, pneumonia, atelectasis, urinary tract infection, and urinary retention.

### 2.1. Statistical Analyses

*R* software (version 3.6.1) was used for statistical descriptions and statistical analyses. Student's *t*-test was used to compare continuous variables between the transfusion and nontransfusion groups, and the chi-square test or Fisher's exact method was used for categorical variables. To incorporate all possible factors for further analyses, factors with a *P* < 0.2 in the univariate analysis were enrolled in the least absolute shrinkage and selection operator (LASSO) analysis. Based on the significant variables in the LASSO analysis, multivariate logistic regression was performed to identify independent risk factors, and the forward LR was used for the variable selection. Afterward, a nomogram was established based on independent risk factors, and the risk score of each patient was calculated. To quantify the predictive ability of the nomogram, the area under the curve (AUC), and the receiver operating characteristic curve (ROC) were used to evaluate the discrimination. The comparison of discrimination between the nomogram and all single risk factors was performed with the “pROC” package. In addition, the calibration curve was used to evaluate the calibration of the nomogram, and decision curve analysis (DCA) was used to estimate the clinical usefulness of the nomogram by calculating the net benefits at different threshold probabilities [19].

Furthermore, Student's *t*-test, the Mann–Whitney rank sum test, the chi-square test, and Fisher's exact test were used to compare the variables between patients with and without 30-day or 90-day complications. Factors with a *P* < 0.1 in the univariate analysis were enrolled in the multivariate logistic regression, and the forward LR was used for the variable selection. Except for special instructions, a *P* value<0.05 (two-sided) was considered significant in the present study.

## 3. Results

### 3.1. Baseline of Patients

A total of 1635 patients met the inclusion criteria during the study period. The average age of the cohort was 57.55 ± 11.38 years, and the average BMI was 25.04 ± 3.67 kg/m^2^. The baseline of all patients is shown in Table 1.

### 3.2. Incidence and Risk Factors for Transfusion

Two hundred and fifty-eight patients (15.78%) were enrolled in the transfusion group, and the remaining 1377 patients were enrolled in the nontransfusion group. In the univariate analysis, the results showed that gender, height, weight, BMI, smoking, alcoholism, indications, anesthesia, operation time, procedures, intraoperative blood loss, TXA use, coronary heart disease, Hb, HCT, RDW, WBC, and RET were significantly different between the transfusion and nontransfusion groups (Table 1). Factors with a *P* value <0.2 in the univariate analysis were included in the LASSO regression analysis to avoid overfitting, and 16 variables were identified as significant factors (Supplementary Figure 1). Then, the significant variables in the LASSO regression analysis were included in the multivariate logistic regression analysis, and the results showed that longer operation time, simultaneous bilateral THA, greater blood loss, comorbid coronary heart disease, lower preoperative Hb, lower preoperative PLT, and lower weight were independent risk factors for transfusion after THA in nonfracture patients. In addition, TXA use, nongeneral anesthesia, and smoking were protective factors for blood transfusion after THA in nonfracture patients (Table 2).

### 3.3. Development of a Nomogram for Transfusion

**Figure 1:**
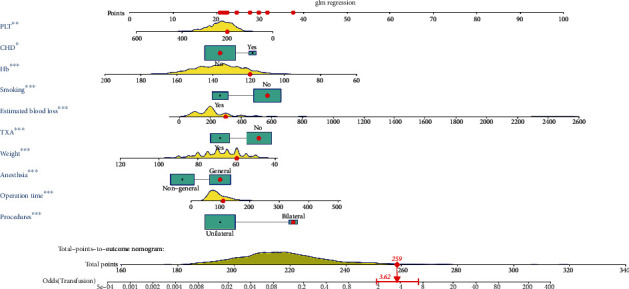
A nomogram based on the ten independent predictors of transfusion. ^∗^*P* < 0.001; ^∗∗^*P* < 0.01; ^∗∗∗^*P* < 0.05.

**Figure 2:**
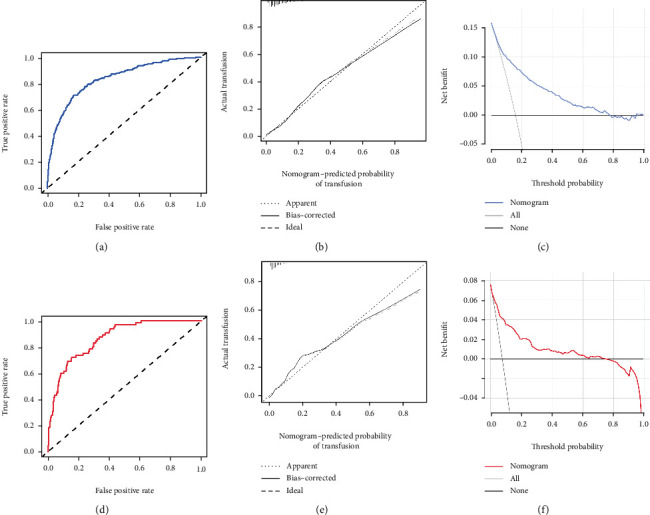
The receiver operating characteristic curve (a), calibration curve (b), and decision curve analysis (c) of training set. The receiver operating characteristic curve (d), calibration curve (e), and decision curve analysis (f) of validation cohort.

Ten independent predictors determined in the present study were selected to establish the nomogram (Figure 1). The AUC of the nomogram was 0.834 (95%CI = 0.807 − 0.862) (Figure 2(a)), which showed good accuracy in predicting transfusion in nonhip fracture patients who underwent THA. The favorable calibration plot of our nomogram indicated that the prediction by the nomogram was highly consistent with the actual observation (Figure 2(b)). Moreover, if the threshold probability of a patient and a doctor was >3 and <78%, respectively, using this nomogram to predict transfusion risk added more benefit to the scheme (Figure 2(c)).

### 3.4. Validation of the Nomogram for Transfusion

From August 2017 to May 2019, 859 patients who met the criteria were prospectively included in the validation cohort (Supplementary Table 1). The transfusion rate in the validation cohort was 7.6% (65/859), which was significantly lower than that in the primary cohort (*χ*^2^ = 33.693, *P* < 0.001). The AUC of the nomogram in predicting transfusion in the validation cohort was 0.867 (95% CI: 0.828-0.907) (Figure 2(d)). In addition, the calibration curve was also plotted. Although the coincidence degree of three line segments was not as high as that of the primary cohort, and this model still had a good calibration in the external validation cohort (Figure 2(e)). The decision curve analysis demonstrated that if the threshold probability was higher than 4% but less than 76%, using the nomogram to predict perioperative transfusion added more net benefit to the scheme (Figure 2(f)).

### 3.5. Comparison of AUC between the Nomogram and a Single Factor

**Figure 3:**
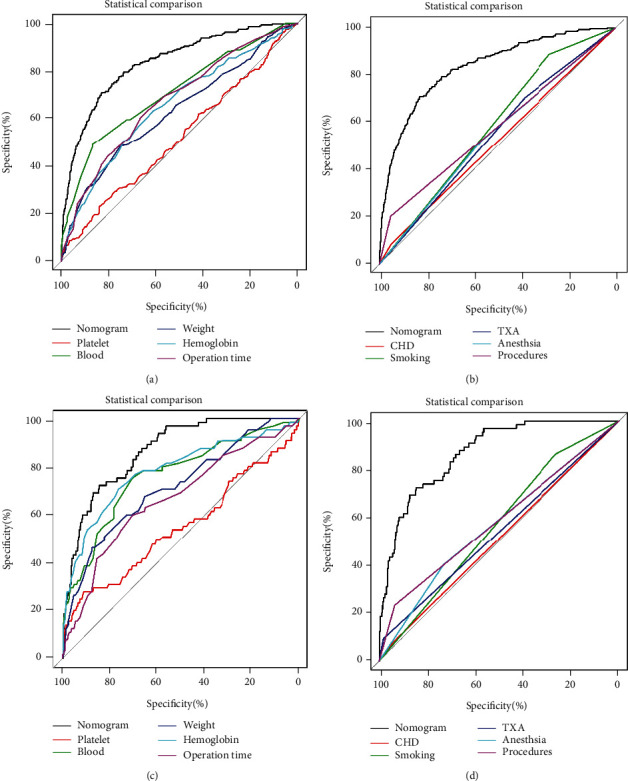
The receiver operating characteristic curve nomogram and independent predictors in training cohort (a, b) and validation cohort (c, d).

The ROC curves of the nomogram, estimated blood loss, operation time, hemoglobin, weight, smoking, procedures, anesthesia, TXA, PLT, and coronary heart disease are shown in Figure 3. In the training cohort, the results demonstrated that the AUC of the nomogram was significantly higher than the AUCs of all single factors in predicting postoperative transfusion in nonhip fracture patients who underwent THA (Figures 3(a) and 3(b)). Moreover, the comparison of AUC in the validation cohort indicated that the AUC of the nomogram was significantly higher than the AUCs of any independent predictors (Figures 3(c) and 3(d)).

### 3.6. The Effects of Transfusion in Patients with Nonhip Fracture after THA

The effects of blood transfusion on patients with nonhip fracture after THA are shown in Table 3. The results showed that the patients who received transfusion had a longer length of hospital stay and higher total in-hospital costs. In addition, a total of 49 patients experienced complications within 30 days after THA, and 61 patients underwent 90-day complications. Detailed information about postoperative complications is shown in Supplementary Table 2. More importantly, the results showed that the patients received transfusion and had a higher incidence of 30- and 90-day complications (Table 3).

### 3.7. Transfusion Is an Independent Risk Factor for Postoperative Complications

**Figure 4:**
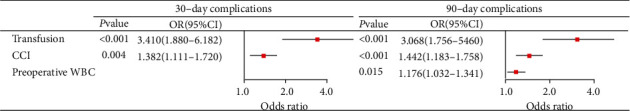
The figure shows the independent risk factors of 30-day and 90-day complications, which indicate that perioperative transfusion is independent risk factor in both 30-day and 90-day complications.

The incidence of complications in patients with nonhip fracture within postoperative days 30 and 90 was 3.00% and 3.73%, respectively. The results of the univariate analysis are presented in Table 4. Factors with *P* < 0.1 in univariate analysis were incorporated into the multivariate logistic regression analysis to determine the independent risk factors for complications. The results showed that the independent risk factors for 30-day complications were perioperative transfusion and CCI (Figure 4). The independent risk factors for 90-day complications included transfusion, CCI, and preoperative WBC (Figure 4).

## 4. Discussion

Blood loss is an unavoidable problem in THA patients, which reduces the Hb level of patients. Allogeneic blood transfusion is a way to increase Hb levels. However, several studies have found that transfusion increases the risk of postoperative complications and mortality as well as costs and length of hospital stay [3–9, 15]. Therefore, it is important to identify high-risk patients and intervene early to reduce the incidence of transfusion. Previous studies have reported the incidence, risk factors, and effects of transfusion in patients undergoing THA. However, to the best of our knowledge, this is the first study to investigate the incidence, risk factors, and effects of transfusion in nonhip fracture patients following THA. The results showed that the incidence of perioperative blood transfusion was 15.78%. Lower weight, general anesthesia, longer operation time, greater intraoperative blood loss, simultaneous bilateral THA, no TXA use, comorbid coronary heart disease, lower preoperative Hb, and lower preoperative PLT were independent risk factors for perioperative blood transfusion in nonhip fracture patients following THA. In addition, a nomogram was established to predict the transfusion risk, and the AUC of our nomogram was 0.834 (95%CI = 0.807 − 0.862). In addition, this study also showed that the total in-hospital costs and length of hospital stay in transfusion patients were significantly higher than those for nontransfusion patients, and perioperative transfusion was an independent risk factor for both 30-day and 90-day complications.

The incidence of postoperative blood transfusion in THA patients reported in previous studies varies, which may be caused by population differences or different blood transfusion policies in different studies. However, several risk factors have been confirmed by numerous studies, such as female sex, lower body weight, longer operation time, simultaneous bilateral THA, no TXA use, and lower preoperative Hb level [1, 5, 20, 21]. In addition to the above risk factors, we found that the incidence of transfusion in patients with general anesthesia was higher than that in patients with nongeneral anesthesia. Previous studies have shown that blood loss was higher in patients who underwent general anesthesia than in patients under local anesthesia during hip arthroplasty [22]. In addition, the operation time was longer with local anesthesia, which may lead to an increased risk of blood transfusion [23]. Blood loss as a risk factor for postoperative transfusion was also confirmed in this study. The relationship between preoperative PLT and the risk of transfusion has also received less attention in previous studies. As an important component of the coagulation system, PLT plays an important role in reducing perioperative blood loss. Therefore, for high-risk patients, preoperative correction of platelet count should be considered.

Based on the nomogram, multiple risk factors can be combined to predict the probability of outcomes and visualize the results. Nomograms are currently widely used in clinical prediction, and many nomograms have been established to predict the inpatient status after THA. In this study, we found that the nomogram based on ten predictors can accurately predict postoperative transfusion risk in nonhip fracture patients following THA. The AUC of the nomogram was 0.834 (95%CI = 0.807 − 0.862), which was higher than the threshold for good performance (AUC > 0.8). In addition, the AUC by the nomogram was significantly higher than the AUCs of all independent predictors. According to the evaluation results, the perioperative management strategy is specified to reduce the risk of transfusion and unnecessary preventive measures to reduce the economic burden and the risk of side effects. More importantly, the nomogram was validated by an independent cohort, and the results of validation confirmed that this nomogram can perform well in predicting perioperative transfusion in nonhip fracture patients following THA.

The relationship between transfusion and postoperative complications at 30 and 90 days was also confirmed in our study. Previous studies have shown that transfusion can increase the risk of deep venous thromboembolism, surgical site infection, periprosthetic joint infection, and mortality following arthroplasty [5–9]. M. A. et al. included 1832 patients following THA, and the results showed that perioperative transfusion was an independent risk factor for postoperative surgical site infection [9]. The relationship between surgical site infection and transfusion in patients with arthroplasty was also confirmed in the study performed by Frisch et al. [5]. Recently, Jiang et al. [6] retrospectively studied 715 patients with lower limb arthroplasty and found that perioperative allogenic blood transfusion was significantly associated with deep venous thromboembolism following total joint arthroplasty. In addition, Browne et al. investigated 129,901 patients with THA and found that transfusion was not only associated with postoperative complications but also closely related to postoperative mortality [24]. However, no study has focused on nonfracture patients. Due to the sample size, we did not study the relationship between specific complications and blood transfusion. However, we found that postoperative blood transfusion was significantly associated with 30-day complications and 90-day complications, which is important to guide clinical practice. The reason for the relationship between the transfusion and adverse events is not only that the blood transfusion is directly attributable to transfusion-associated cardiac overload or transfusion-related lung injury [25] but also that the higher intraoperative bleeding and longer length of hospital stay in patients with blood transfusion may be important reasons.

In addition, the results of this study indicated that the total in-hospital costs in the transfusion group were higher than those in the nontransfusion group. The increase in costs may be due to a series of costs arising from the use of blood products. In addition, the increase in hospitalization days and the proportion of complications among transfusion patients may also be a reason for the increase in costs. In this study, we also found that the length of hospital stay in the transfusion group was higher than that in the nontransfusion group. The increase in hospitalization time for blood transfusion has been confirmed by a large number of studies [24, 26, 27]. In this study, the average hospitalization time of patients in the transfusion group was nearly 2 days longer than that of nontransfusion patients.

This study also has some limitations. First, due to the short follow-up period, the incidence of complications may be underestimated. In addition, complications were not classified in this research, and the relationship between blood transfusion and specific complications could not be determined. Second, this study was a single-center, retrospective study, which has the inherent bias possibility of such research, and the nomogram was not externally validated. Finally, due to the relatively small sample size of patients in the transfusion group, the relationship between different transfusion volumes and postoperative complications was not studied in this study. In the future, multicenter, prospective studies are needed to better demonstrate this conclusion.

## 5. Conclusion

The transfusion rate of nonfracture patients within 14 days after THA was 15.78%. Lower weight, general anesthesia, longer operation time, simultaneous bilateral THA, no TXA use, comorbid coronary heart disease, lower preoperative Hb, and lower preoperative PLT were risk factors for postoperative blood transfusion. A validated nomogram was established based on ten common variables for transfusion after THA. Among the patients who underwent THA for nonfracture diseases, the total in-hospital costs and the length of hospital stay in the transfusion group were higher than those in the nontransfusion group, and blood transfusion was an independent risk factor for complications within 30 days and 90 days.

## Figures and Tables

**Table 1:** Univariate analysis of blood transfusion in nonfracture patients after total hip arthroplasty.

	Total (*n* = 1635)	Transfusion (*n* = 258)	Nontransfusion (*n* = 1377)	*P*
Age, years	57.55 ± 11.38	57.29 ± 12.67	57.59 ± 11.12	0.716
Gender (male)	942 (57.6)	106 (11.3)	836 (88.7)	<0.001
Height, cm	165.45 ± 7.66	163.49 ± 7.66	165.82 ± 7.60	<0.001
Weight, kg	68.57 ± 11.32	64.30 ± 11.61	69.37 ± 11.08	<0.001
BMI, kg/㎡	25.04 ± 3.67	24.02 ± 3.77	25.23 ± 3.62	<0.001
Blood type				0.236
A	519 (31.7)	70 (13.5)	449 (86.5)	
B	515 (31.5)	84 (16.3)	431 (83.7)	
O	439 (26.9)	80 (18.2)	359 (81.8)	
AB	162 (9.9)	24 (14.8)	138 (85.2)	
Smoking	413 (25.3)	29 (7.0)	384 (93.0)	<0.001
Alcoholism	438 (26.8)	42 (9.6)	396 (90.4)	<0.001
CCI	1.73 ± 1.20	1.83 ± 1.32	1.71 ± 1.18	0.174
Indications				0.041
ANFH	1282 (78.4)	189 (14.7)	1093 (85.3)	
OA	162 (9.9)	28 (17.3)	134 (82.7)	
RA	8 (0.5)	3 (37.5)	5 (62.5)	
AS	9 (0.6)	4 (44.4)	5 (55.6)	
DDH	169 (10.3)	33 (19.5)	136 (80.5)	
Others	5 (0.3)	1 (20.0)	4 (80.0)	
Anesthesia				0.007
General	731 (44.7)	135 (18.5)	596 (81.5)	
Nongeneral	904 (55.3)	123 (13.6)	781 (86.4)	
Operation time, min	97.97 ± 45.31	122.95 ± 61.80	93.28 ± 39.82	<0.001
Procedures				<0.001
Unilateral	1518 (92.8)	206 (13.6)	1312 (86.4)	
Bilateral	117 (7.2)	52 (44.4)	65 (55.6)	
Approach				0.903
Anterior	17 (1.0)	2 (11.8)	15 (88.2)	
Posterior	1618 (99.0)	256 (15.8)	1362 (84.2)	
Estimated blood loss, ml	263.69 ± 212.70	429.26 ± 360.23	232.67 ± 152.92	<0.001
Drainage use	342 (20.9)	45 (13.2)	297 (86.8)	0.135
TXA use	604 (36.9)	76 (12.6)	528 (87.4)	0.007
Comorbidities				
Hypertension	432 (26.4)	65 (15.0)	367 (85.0)	0.626
Diabetes	116 (7.1)	21 (18.1)	95 (81.9)	0.476
Coronary heart disease	91 (5.6)	22 (24.2)	69 (75.8)	0.024
Cerebrovascular diseases	75 (4.6)	11 (14.7)	64 (85.3)	0.787
Arrhythmia	17 (1.0)	5 (29.4)	12 (70.6)	0.224
Respiratory diseases	69 (4.2)	12 (17.4)	57 (82.6)	0.708
Digestive diseases	98 (6.0)	16 (16.3)	82 (83.7)	0.878
Urinary diseases	35 (2.1)	5 (14.3)	30 (85.7)	0.806
Mental diseases	15 (0.9)	2 (13.3)	13 (86.7)	1.000
Fibroid	28 (1.7)	2 (7.1)	26 (92.9)	0.206
Preoperative Hb, g/L	135.96 ± 15.41	128.66 ± 17.37	137.33 ± 14.62	<0.001
Preoperative HCT, %	40.57 ± 4.07	38.66 ± 4.48	40.92 ± 3.89	<0.001
Preoperative RDW, %	13.14 ± 1.03	13.30 ± 1.26	13.11 ± 0.98	0.021
Preoperative WBC, n/L	6.30 ± 1.76	5.89 ± 1.73	6.37 ± 1.75	<0.001
Preoperative PLT, n/L	223.82 ± 60.70	227.40 ± 64.56	233.85 ± 59.92	0.117
Preoperative RET, %	0.06 ± 0.02	0.05 ± 0.02	0.06 ± 0.02	<0.001

BMI: body mass index; CCI: Charlson comorbidity index; ANFH: avascular necrosis of femoral head; OA: osteoarthritis; RA: rheumatoid arthritis; AS: ankylosing spondylitis; DDH: developmental dysplasia of the hip; TXA: tranexamic acid; Hb: hemoglobin; HCT: hematocrit; RDW: red cell distribution width; WBC: white blood cell; PLT: platelet; RET: reticulocyte.

**Table 2:** Multivariate logistic analysis of transfusion in nonfracture patients after total hip arthroplasty.

	Estimated parameter	SE	Wald	95% CI	*P*
Weight	-0.038	0.008	25.537	0.948-0.977	<0.001
Smoking (yes)	-0.942	0.248	14.386	0.240-0.634	<0.001
Anesthesia (non-general)	-0.743	0.165	20.255	0.344-0.657	<0.001
Procedures (bilateral)	1.450	0.305	22.653	2.346-7.742	<0.001
Operation time	0.006	0.002	7.932	1.002-1.010	0.005
Estimated blood loss	0.003	0.000	60.863	1.002-1.004	<0.001
Tranexamic acid (use)	-0.773	0.189	16.642	0.318-0.669	<0.001
Coronary heart disease (yes)	0.650	0.291	4.988	1.083-3.391	0.026
Platelet	-0.004	0.001	11.276	0.993-0.998	0.001
Hemoglobin	-0.035	0.006	40.339	0.955-0.976	<0.001

SE: standard error; OR: odds ratio; CI: confidence interval.

**Table 3:** The effect of blood transfusion in nonfracture patients after total hip arthroplasty.

	Transfusion (*n* = 258)	Nontransfusion (*n* = 1377)	*P*
Length of hospital stay	10.73 ± 4.79	8.89 ± 2.49	<0.001
Total in-hospital costs	79319.94 ± 27462.30	64651.16 ± 17383.79	<0.001
30-day complications	19 (7.4)	30 (2.2)	<0.001
90-day complications	21 (8.1)	40 (2.9)	<0.001

**Table 4:** Univariate analysis of postoperative complications in nonhip fracture patients after total hip arthroplasty.

	30-day complications			90-day complications		
	Complication (*n* = 49)	Noncomplication (*n* = 1586)	*P*	Complication (*n* = 61)	Noncomplication (*n* = 1574)	*P*
Transfusion			<0.001			<0.001
Yes	19 (38.8)	239 (15.1)		21 (34.4)	237 (15.1)	
No	30 (61.2)	1347 (84.9)		40 (65.6)	1337 (84.9)	
Age, years	61.59 ± 10.72	57.42 ± 11.38	0.011	61.56 ± 10.50	57.39 ± 11.39	0.005
Gender (M)	27 (55.1)	915 (57.7)	0.718	33 (54.1)	909 (57.8)	0.571
BMI, km/㎡	25.10 ± 3.61	25.03 ± 3.67	0.902	25.13 ± 3.46	25.03 ± 3.68	0.830
CCI	2.27 ± 1.38	1.72 ± 1.19	0.002	2.30 ± 1.42	1.71 ± 1.19	<0.001
Indications			0.774			0.423
ANFH	40 (81.6)	1242 (78.3)		50 (82.0)	1232 (78.3)	
OA	6 (12.2)	156 (9.8)		6 (9.8)	156 (9.9)	
RA	0 (0.0)	8 (0.5)		0 (0.0)	8 (0.5)	
AS	0 (0.0)	9 (0.6)		0 (0.0)	9 (0.6)	
DDH	3 (6.1)	166 (10.5)		4 (6.6)	165 (10.5)	
Others	0 (0.0)	5 (0.3)		1 (1.6)	4 (0.3)	
Smoking	9 (18.4)	404 (25.5)	0.260	402 (25.5)	11 (18.0)	0.185
Drinking	13 (26.5)	425 (26.8)	0.967	422 (26.8)	16 (26.2)	0.920
Anesthesia			0.254			0.262
General	18 (36.7)	713 (45.0)		23 (37.7)	708 (45.0)	
Nongeneral	31 (63.3)	873 (55.0)		38 (62.3)	866 (55.0)	
Operation time, min	99.37 ± 41.68	97.92 ± 45.43	0.826	103.76 ± 43.18	97.74 ± 45.39	0.309
Blood loss, ml	303.47 ± 268.11	262.42 ± 210.75	0.294	297.87 ± 258.38	262.37 ± 210.72	0.293
Procedures			0.776			0.661
Unilateral	46 (93.9)	1472 (92.8)		58 (95.1)	1460 (92.8)	
Bilateral	3 (6.1)	114 (7.2)		3 (4.9)	114 (7.2)	
Approach			0.466			1.000
Anterior	0 (0.0)	17 (1.1)		0 (0.0)	17 (1.1)	
Posterior	49 (100)	1569 (98.9)		61 (100)	1557 (98.9)	
Drainage use	14 (28.6)	328 (20.7)	0.181	16 (26.2)	326 (20.7)	0.298
TXA use	13 (26.5)	591 (37.3)	0.125	18 (29.5)	586 (37.2)	0.220
Complication						
Hypertension	17 (34.7)	415 (26.2)	0.182	22 (36.1)	410 (26.2)	0.082
Diabetes	4 (8.2)	112 (7.1)	0.989	4 (6.6)	112 (7.1)	1.000
Coronary hear disease	4 (8.2)	87 (5.5)	0.625	6 (9.8)	85 (5.4)	0.231
Cerebrovascular diseases	6 (12.2)	69 (4.4)	0.024	8 (13.1)	67 (4.3)	0.003
Arrhythmia	0 (0.0)	17 (1.1)	1.000	1 (1.6)	16 (1.0)	0.478
Respiratory diseases	3 (6.1)	66 (4.2)	0.755	6 (9.8)	63 (4.0)	0.058
Digestive diseases	5 (10.2)	93 (5.9)	0.340	5 (8.2)	93 (5.9)	0.643
Urinary diseases	3 (6.1)	32 (2.0)	0.146	3 (4.9)	32 (2.0)	0.282
Mental diseases	0 (0.0)	15 (0.9)	1.000	0 (0.0)	15 (1.0)	1.000
Fibroid	1 (2.0)	27 (1.7)	0.576	1 (1.6)	27 (1.7)	1.000
Preoperative Hb, g/L	133.41 ± 14.24	136.04 ± 15.44	0.239	133.11 ± 14.97	136.07 ± 15.42	0.141
Preoperative HCT, %	39.89 ± 3.80	40.59 ± 4.08	0.237	39.78 ± 3.98	40.60 ± 4.07	0.125
Preoperative RDW, %	13.17 ± 0.71	13.13 ± 1.04	0.839	13.25 ± 0.80	13.13 ± 1.04	0.376
Preoperative WBC, n/L	6.53 ± 1.86	6.29 ± 1.76	0.351	6.68 ± 1.95	6.28 ± 1.75	0.083
Preoperative PLT, n/L	229.22 ± 66.16	232.95 ± 60.55	0.673	229.74 ± 64.22	232.95 ± 60.58	0.685
Preoperative RET, 10^12^/L	0.06 ± 0.02	0.06 ± 0.02	0.405	0.06 ± 0.02	0.06 ± 0.02	0.605

BMI: body mass index; CCI: Charlson comorbidity index; ANFH: avascular necrosis of femoral head; OA: osteoarthritis; RA: rheumatoid arthritis; AS: ankylosing spondylitis; DDH: developmental dysplasia of the hip; TXA: tranexamic acid; Hb: hemoglobin; HCT: hematocrit; RDW: red cell distribution width; WBC: white blood cell; PLT: platelet; RET: reticulocyte.

## Data Availability

The datasets generated during and/or analyzed during the current study are available from the corresponding author on reasonable request.

## Supplementary Materials

Supplementary 1 Supplementary Figure 1. the results of LASSO regression analysis.

Supplementary 2 Supplementary Table 1. Comparison of baseline information between training and validation cohorts.

Supplementary 3 Supplementary Table 2. Postoperative complications in non-hip fracture patients after total hip arthroplasty.
