# Supplementary figures and images for: Incidence, Risk Factors, and Nomogram of Transfusion and Associated Complications in Nonfracture Patients following Total Hip Arthroplasty
Source: Biomed Res Int. 2020 Oct 14;2020:2928945. doi: 10.1155/2020/2928945 (PMC7584933; doi:10.1155/2020/2928945)

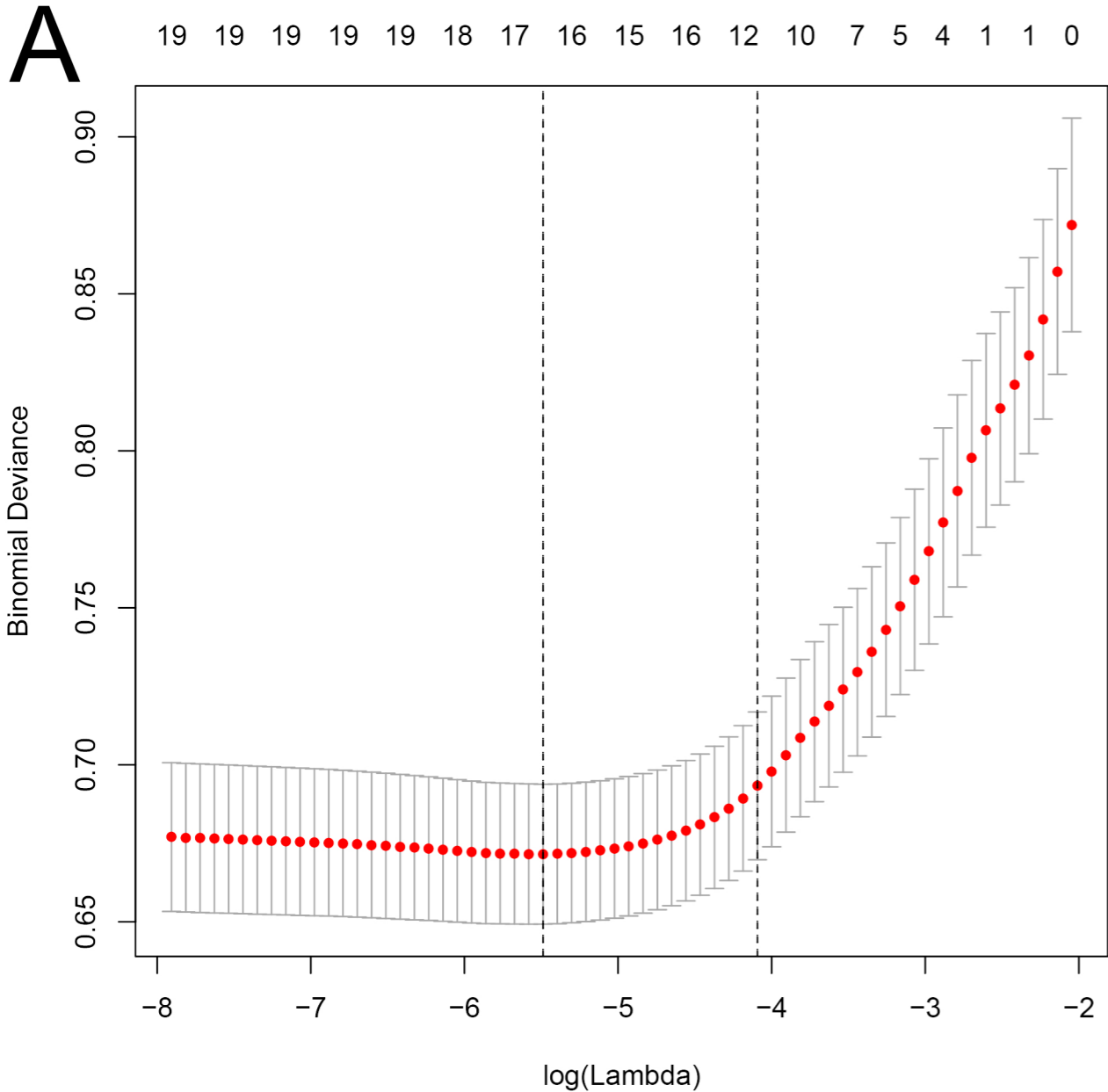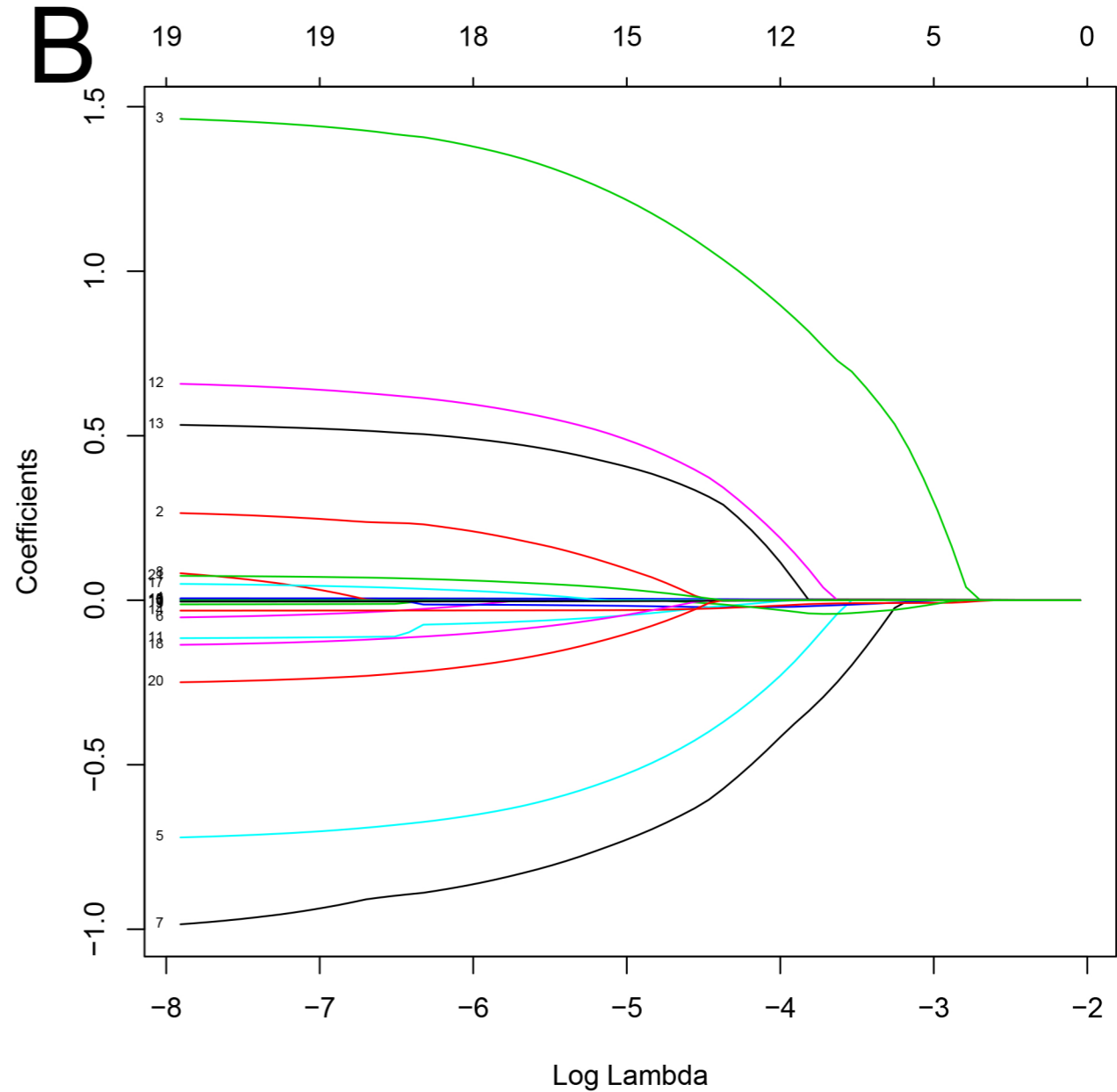

Supplement: Supplementary 1 — Supplementary Figure 1. the results of LASSO regression analysis. [file 2928945.f1.pdf]
