# Supplementary material for: Incidence, Risk Factors, and Nomogram of Transfusion and Associated Complications in Nonfracture Patients following Total Hip Arthroplasty
Source: Biomed Res Int. 2020 Oct 14;2020:2928945. doi: 10.1155/2020/2928945 (PMC7584933; doi:10.1155/2020/2928945)
Supplement: Supplementary 2 — Supplementary Table 1. Comparison of baseline information between training and validation cohorts. [file 2928945.f2.docx]

| Supplementary Table 1. Comparison of baseline information between training and validation cohorts | | | |
| --- | --- | --- | --- |
|  | Training cohort(n=1635) | Validation cohort(n=1635) | P |
| Transfusion | 258(15.8) | 65(7.6) | <0.001 |
| Age, years | 57.55±11.38 | 58.54±10.61 | 0.030 |
| Gender (Male) | 942(57.6) | 519(60.4) | 0.177 |
| Weight, kg | 68.57±11.32 | 68.28±11.49 | 0.539 |
| BMI, kg/㎡ | 25.04±3.67 | 24.92±3.55 | 0.466 |
| Smoking | 413(25.3) | 219(25.5) | 0.898 |
| CCI | 1.73±1.20 | 1.78±1.21 |  |
| Anesthesia |  |  | <0.001 |
| General | 731(44.7) | 239(27.8) |  |
| Non-general | 904(55.3) | 620(72.2) |  |
| Operation time, min | 97.97±45.31 | 90.84±35.06 | <0.001 |
| Procedures |  |  | 0.631 |
| Unilateral | 1518(92.8) | 793(92.4) |  |
| Bilateral | 117(7.2) | 66(7.7) |  |
| Estimated blood loss, ml | 263.69±212.70 | 232.64±161.60 | <0.001 |
| TXA use | 604(36.9) | 839(97.7) | <0.001 |
| Coronary heart disease | 91(5.6) | 51(5.9) | 0.704 |
| Preoperative Hb, g/L | 135.96±15.41 | 138.39±15.38 | <0.001 |
| Preoperative PLT, n/L | 223.82±60.70 | 239.68±69.02 | 0.014 |

BMI: Body mass index, CCI: Charlson comorbidity index, TXA: Tranexamic acid, Hb: Hemoglobin, PLT: Platelet
