# Supplementary material for: Incidence, Risk Factors, and Nomogram of Transfusion and Associated Complications in Nonfracture Patients following Total Hip Arthroplasty
Source: Biomed Res Int. 2020 Oct 14;2020:2928945. doi: 10.1155/2020/2928945 (PMC7584933; doi:10.1155/2020/2928945)
Supplement: Supplementary 3 — Supplementary Table 2. Postoperative complications in non-hip fracture patients after total hip arthroplasty. [file 2928945.f3.docx]

| Supplementary Table 2. Postoperative complications in non-hip fracture patients after total hip arthroplasty | | |
| --- | --- | --- |
|  | 30-day complications(n=49) | 90-day complications(n=61) |
| Pulmonary embolism | 2(4.1) | 2(3.3) |
| Cerebrovascular complication | 2(4.1) | 5(8.2) |
| Cardiovascular complication | 8(16.3) | 8(13.1) |
| Delirium | 3(6.1) | 3(4.9) |
| Digestive complication | 10(20.4) | 10(16.4) |
| Anaphylaxis | 4(8.2) | 4(6.6) |
| Infection | 3(6.1) | 4(6.6) |
| Shock | 2(4.1) | 2(3.3) |
| Respiratory complication | 3(6.1) | 4(6.6) |
| Urological complication | 6(12.2) | 6(9.8) |
| Other | 6(12.2) | 13(21.3) |
